# Pushing the Limits of Biosensing: Selective Calcium Ion Detection with High Sensitivity via High-*k* Gate Dielectric Engineered Si Nanowire Random Network Channel Dual-Gate Field-Effect Transistors

**DOI:** 10.3390/s23156720

**Published:** 2023-07-27

**Authors:** Tae-Hwan Hyun, Won-Ju Cho

**Affiliations:** Department of Electronic Materials Engineering, Kwangwoon University, Seoul 139-701, Republic of Korea; gusxod97@gmail.com

**Keywords:** biosensor, silicon nanowire random network, dual-gate field-effect transistor, self-amplification, calcium ion-selective, extended gate, capacitive coupling, high-*k* gate dielectric engineering

## Abstract

Calcium ions (Ca^2+^) are abundantly present in the human body; they perform essential roles in various biological functions. In this study, we propose a highly sensitive and selective biosensor platform for Ca^2+^ detection, which comprises a dual-gate (DG) field-effect transistor (FET) with a high-*k* engineered gate dielectric, silicon nanowire (SiNW) random network channel, and Ca^2+^-selective extended gate. The SiNW channel device, which was fabricated via the template transfer method, exhibits superior Ca^2+^ sensing characteristics compared to conventional film channel devices. An exceptionally high Ca^2+^ sensitivity of 208.25 mV/dec was achieved through the self-amplification of capacitively coupled DG operation and an enhanced amplification ratio resulting from the high surface-to-volume ratio of the SiNW channel. The SiNW channel device demonstrated stable and reliable sensing characteristics, as evidenced by minimal hysteresis and drift effects, with the hysteresis voltage and drift rate measuring less than 6.53% of the Ca^2+^ sensitivity. Furthermore, the Ca^2+^-selective characteristics of the biosensor platform were elucidated through experiments with pH buffer, NaCl, and KCl solutions, wherein the sensitivities of the interfering ions were below 7.82% compared to the Ca^2+^ sensitivity. The proposed Ca^2+^-selective biosensor platform exhibits exceptional performance and holds great potential in various biosensing fields.

## 1. Introduction

Calcium ions (Ca^2+^) are the most abundant metal ions found in the human body. These ions are responsible for performing various biological functions, such as blood clotting, intercellular adhesion, skeletal integrity maintenance, and cell mobility facilitation. Maintaining appropriate Ca^2+^ levels within the body is critical for sustaining optimal biological health [1,2]. However, high concentrations of Ca^2+^ can be highly toxic, necessitating the precise regulation of physiological Ca^2+^ concentrations within specific limits. Therefore, employing appropriate analytical approaches to determine physiological concentrations of Ca^2+^ is crucial [3,4]. However, the human body comprises other cations, such as Na^+^ and K^+^. Consequently, in most cases, Ca^2+^ must be selectively detected in the presence of other interfering ions. Several studies have focused on designing sensors, such as potentiometric, galvanostatic, and colorimetric sensors, for the selective detection of Ca^2+^ [5,6,7,8].

An ion-sensitive field-effect transistor (ISFET) was first reported in the 1970s [9]. This field-effect transistor (FET)-type sensor system offers numerous advantages, such as fast response, label-free detection, and compatibility with the complementary metal-oxide-semiconductor (CMOS) process [10,11,12,13]. The concept of ISFETs has been further advanced to an extended-gate field-effect transistor (EGFET) structure, which comprises a separated extended gate (EG) sensing unit and an FET transducer unit [14,15,16,17]. By adopting the EG structure, disposable EGs can protect high-cost FETs from chemical damage, because the analyte solution is not in direct contact with the FETs. Despite their many desirable features, conventional single-gate (SG) structured ISFETs suffer from a critical drawback, known as the Nernstian limit, which restricts their sensitivity. According to this theoretical limitation, conventional ISFETs cannot exceed a sensitivity of 59.14 mV/pH at 300 K [18]. Therefore, improving their sensitivity is essential for the wider application of FET-type sensors. The dual-gate (DG) structure, which has capacitively coupled top- and bottom-gate electrodes, can amplify sensitivity itself through its structural features. Additionally, employing a high-*k* gate dielectric engineered DG structure, which replaces the top-gate insulator with a high-*k* insulator, can be employed to further improve the sensitivity of ISFETs by increasing the top-gate insulator capacitance [19,20,21,22]. Consequently, constructing DG-structured FET-type sensors is an effective and promising approach for developing highly sensitive sensor platforms that can overcome the Nernstian limit. 

Silicon nanowire (SiNW) channel-based FET-type sensors have recently attracted attention as promising biosensor platforms owing to their advantageous properties such as superior sensitivity, high selectivity, and wide applicability. In recent years, numerous studies have reported various applications of SiNW channel biosensors, including detection of pH, chemicals, neurotransmitters, DNA, proteins, and viruses [23,24,25,26,27]. The high surface-to-volume ratio of the SiNW channel enhances gate capacitance and gate controllability, providing excellent charge control and operational performance [23]. Furthermore, in DG structured ISFETs, SiNW channel provides higher top-gate oxide capacitance, which results in a higher amplification of the sensitivity. However, the conventional formation process of the SiNW channels generally requires complex and expensive procedures such as vapor–liquid–solid (VLS) growth, plasma-enhanced chemical deposition (PECVD), electron beam lithography, and deep ultraviolet (DUV) photolithography [28,29,30]. Meanwhile, the template transfer method allows for the fabrication of SiNW random network channels through simpler processes such as electrospinning and reactive ion etching (RIE), which are commonly used in CMOS processing. By employing the template transfer method to create a SiNW random network channel, it is possible to easily achieve the advantages of a high surface-to-volume ratio and high gate capacitance of the SiNW channels.

In this study, we propose a high-performance Ca^2+^-selective biosensor platform based on high-*k* gate dielectric engineered SiNW random network channel DG FETs. The SiNW channel was fabricated using a template transfer method, utilizing polyvinylpyrrolidone (PVP) nanofibers as the pattern template. The electrical and sensing properties of the fabricated high-*k* gate dielectric engineered SiNW channel DG FETs, including the transfer curves, output curves, pH sensing, and Ca^2+^-selective sensing characteristics, were elucidated. These properties were compared to those of conventional film channel devices. Owing to the advantageous high surface-to-volume ratio of the SiNW channel, the SiNW channel device exhibited a superior sensing performance, including significantly improved self-amplification capability, sensitivity, and stability, than that of the film channel device. Therefore, the proposed high-performance Ca^2+^-selective biosensor based on high-*k* gate dielectric engineered SiNW random network channel DG FETs holds great promise as a sensor platform with exceptional sensitivity, remarkable selectivity, and reliable sensing characteristics, thereby enabling a wide range of applications in various biosensing fields.

## 2. Materials and Methods

### 2.1. Materials

The following materials were used in this study: SiO_2_ sputter target (purity ≥ 99.99%, THIFINE Co., Ltd., Incheon, Republic of Korea), glass substrates (7059 glass; Corning Inc., Corning, NY, USA), Ta_2_O_5_ sputter target (purity ≥ 99.99%, THIFINE Co., Ltd., Incheon, Republic of Korea), indium tin oxide (ITO) sputter target (purity ≥ 99.99%, THIFINE Co., Ltd.), SnO_2_ sputter target (purity ≥ 99.99%, THIFINE Co., Ltd.), phosphosilicate glass (PSG; Filmtronics Inc., Butler, PA, USA), 30:1 buffered oxide etchant (BOE; J.T. Baker, Phillipsburg, NJ, USA), pH buffer solution (Samchun Chemical, Pyeongtack, Republic of Korea), ethanol (Samchun Chemical), polydimethylsiloxane (PDMS; Sylgard 184 silicon elastomer; Dow Corning, Midland, MI, USA), deionized water (DI water; conductivity ≤ 4.3 μS/cm, Sigma-Aldrich, St. Louis, MO, USA), phosphate-buffered saline (PBS; pH 7.4, Sigma-Aldrich), Ca ionophore IV (C_52_H_100_N_2_O_3_, Sigma-Aldrich), 2-nitrophenyl octyl ether (purity ≥ 99.0%, Sigma-Aldrich), polyvinyl chloride (PVC, Sigma-Aldrich), sodium tetrakis [3,5-bis(trifluoromethyl)phenyl]borate (Na-TFPB, Sigma-Aldrich), tetrahydrofuran (THF, Sigma-Aldrich), sodium chloride (NaCl, Sigma-Aldrich), calcium chloride (CaCl_2_, Sigma-Aldrich), and potassium chloride (KCl, Sigma-Aldrich). All the materials were used without any further purification.

We prepared CaCl_2_, NaCl, and KCl solutions by dissolving CaCl_2_, NaCl, and KCl, respectively, in DI water. Through sequential dilution, we prepared solutions with concentrations of 10^0^ M, 10^−1^ M, 10^−2^ M, 10^−3^ M, and 10^−4^ M for each ion.

### 2.2. Formation of SiNW Random Network Channel via the Template Transfer Method

**Figure 1:**
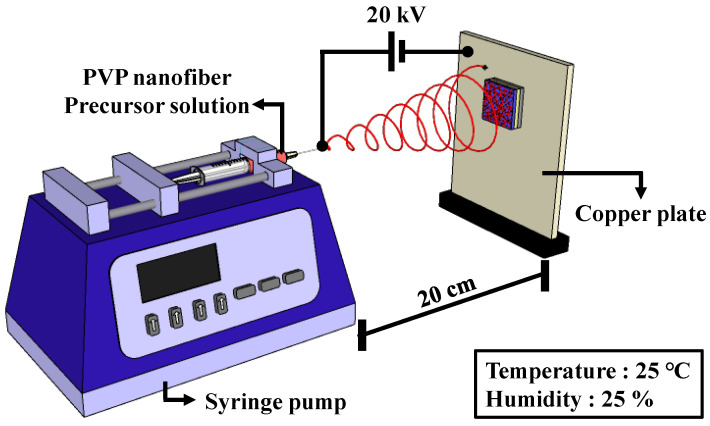
Schematic of the electrospinning system. The electrospinning process was conducted under controlled conditions with a humidity of 25% and temperature of 25 °C.

A SiNW random network channel was obtained using a template transfer method employing electrospun PVP nanofibers. This method offers a straightforward approach for creating a SiNW random network structure through electrospinning and RIE. The formation process of the SiNW channel commenced with the preparation of a silicon-on-insulator (SOI) substrate with defined active regions and n-doped source/drain (S/D) electrodes, as described in Section 2.3. To confine the SiNW pattern to the channel area, the S/D regions were protected by depositing a 200 nm SiO_2_ layer, which was patterned using a lift-off process. Subsequently, PVP nanofibers were deposited as a pattern template via an electrospinning process utilizing a PVP precursor solution under a controlled humidity of 25% and room temperature (25 °C). The PVP precursor solution was prepared by dissolving 200 mg of PVP in 3 mL of ethanol, followed by stirring at 800 RPM for 4 h at room temperature. Figure 1 shows a schematic of the electrospinning system.

**Figure 2:**
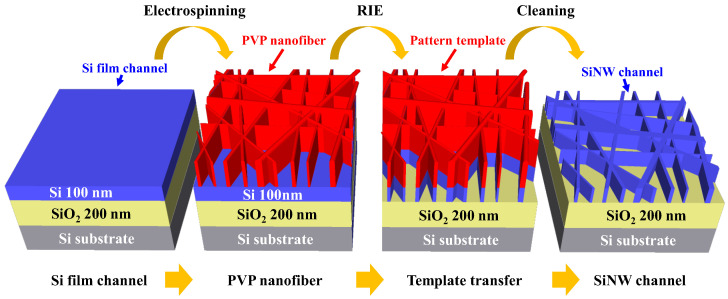
Process flow of the template transfer method using electrospun PVP nanofibers.

After the electrospinning process, conventional thermal annealing (CTA) was performed in a furnace system at 300 °C, which is the melting point of PVP nanofibers. This step was performed to solidify the patterned template and enhance its adhesion to the Si film. The pattern template of the PVP nanofibers was then transferred onto the underlying Si channel layer through RIE in an SF_6_ plasma ambient. Any residual PVP nanofibers were removed via wet etching using a sulfuric acid–hydrogen peroxide mixture. Finally, the shielding oxide layers were removed using a 30:1 buffered oxide etchant (BOE). Figure 2 illustrates the process flow of the template transfer method using electrospun PVP nanofibers.

### 2.3. Fabrication of the SiNW DG FET Transducer Unit

**Figure 3:**
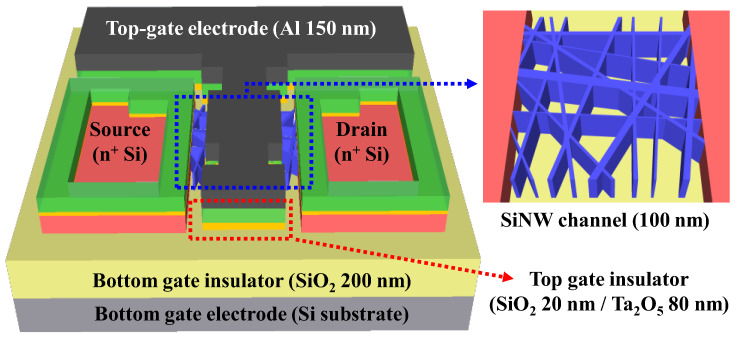
Schematic of the fabricated SiNW DG FET transducer unit.

A 1 × 1 cm^2^ p-type (100) SOI substrate with a 100 nm thick top silicon layer and 200 nm thick buried oxide (BOX) layer was prepared. The resistivity and boron doping concentration of the top silicon layer were 1–10 Ω·cm and 1 × 10^15^ cm^−3^, respectively. To eliminate surface impurities and contaminants, the substrate was cleaned via a standard Radio Corporation of America (RCA) cleaning process. Active regions with a channel layer width/length of 130/90 μm were formed using photolithography and RIE. A 200 nm thick SiO_2_ layer was blanket-deposited using RF magnetron sputtering as a dummy oxide for the phosphorus doping process. The source and drain (S/D) areas were patterned using photolithography, followed by the use of 30:1 BOE to etch the dummy oxide on the S/D area. For n+ doping of the S/D regions, a PSG film was spin-coated and thermally diffused using a rapid thermal annealing (RTA) process at 950 °C for 30 s in an O_2_/N_2_ ambient. The residual PSG and dummy oxide layers were removed using 30:1 BOE. Next, the SiNW channel formation process was performed, as described in Section 2.2. After the SiNW channel was formed, a 20 nm thick SiO_2_ layer and an 80 nm thick Ta_2_O_5_ layer were deposited as high-*k* engineered top-gate oxides using RF magnetron sputtering and a lift-off process. A top-gate electrode of 150 nm thick Al was formed using an electron-beam evaporator and the lift-off process. Simultaneously, a film channel DG FET was fabricated as a reference device without SiNW channel formation. To enhance the overall electrical properties of the fabricated devices, a forming gas annealing (FGA) process was performed at 450 °C for 30 min in a 2% H_2_/N_2_ atmosphere in a furnace. Figure 3 shows a schematic of the fabricated SiNW DG FET transducer unit.

### 2.4. Fabrication of Ca^*2*+^-Selective EG Sensing Unit

**Figure 4:**
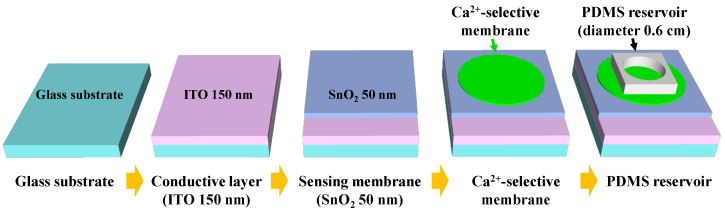
Fabrication flow of the Ca^2+^-selective EG sensing unit.

The EG sensing unit was fabricated on a glass substrate (1.5 cm × 2.5 cm). A 300 nm thick ITO layer was deposited as the conductive layer, which was electrically connected to the top-gate electrode of the transducer through an electrical cable. Subsequently, a 50 nm thick layer of SnO_2_ was deposited as a sensing membrane. The SnO_2_ sensing membrane transfers the surface potential of the analyte solution to the transducer unit via the ITO conductive layer. The ITO and SnO_2_ layers were deposited using an RF magnetron sputtering system. To form a Ca^2+^-selective membrane, a Ca^2+^-selective cocktail (100 μL) was drop-casted onto the SnO_2_ sensing layer and allowed to dry in ambient air at room temperature for 24 h to evaporate the solvent and enhance adhesion. The Ca^2+^-selective cocktail was prepared by dissolving 1.2 mg of Ca ionophore IV, 0.3 mg of Na-TFPB, 32.5 mg of PVC, and 66 mg of 2-nitrophenyl octyl ether in 660 μL of THF. The mixture was then stirred at 800 RPM for 6 h at room temperature. Finally, a sensing region with a diameter of 0.6 cm was defined by attaching a PDMS reservoir to the center of the Ca^2+^-selective membrane. The process flow of the Ca^2+^-selective EG sensing unit is illustrated in Figure 4.

### 2.5. Device Characterization

**Figure 5:**
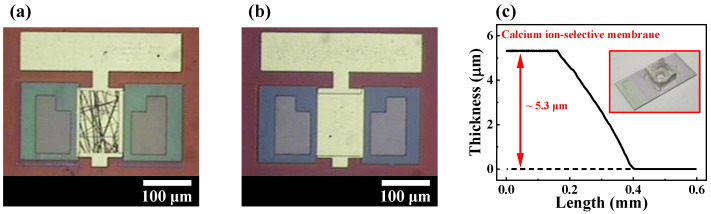
Optical microscopic images of the fabricated high-*k* dielectric engineered (**a**) SiNW random network channel and (**b**) film channel DG FETs. (**c**) Thickness of the drop-casted Ca^2+^-selective membrane. The inset image is a photograph of the fabricated EG sensing unit.

The thicknesses of Si, Al, SiO_2_, Ta_2_O_5_, SnO_2_, ITO, and drop-casted Ca^2+^-selective membranes were measured using a Dektak XT Bruker stylus profiler (Bruker, Hamburg, Germany). The electrical characteristics of the devices were measured using an Agilent 4156 B precision semiconductor parameter analyzer (Agilent Technologies, Santa Clara, CA, USA). A commercial Ag/AgCl electrode (Horiba 2086A-06T, Kyoto, Japan) was used as the reference electrode for pH and Ca^2+^-selective sensor platforms. To minimize external interference, all the electrical measurements were performed in an electromagnetically shielded dark box. Figure 5a,b shows the optical microscopic images of the fabricated high-*k* gate dielectric engineered SiNW and film channel DG FET, respectively. The thickness of the Ca^2+^-selective membrane was approximately 5.3 μm, as depicted in Figure 5c. The inset of Figure 5c shows a photograph of the fabricated EG sensing unit.

## 3. Results

### 3.1. Electrical Characteristics of High-k Gate Dielectric Engineered SiNW Channel DG FETs

**Figure 6:**
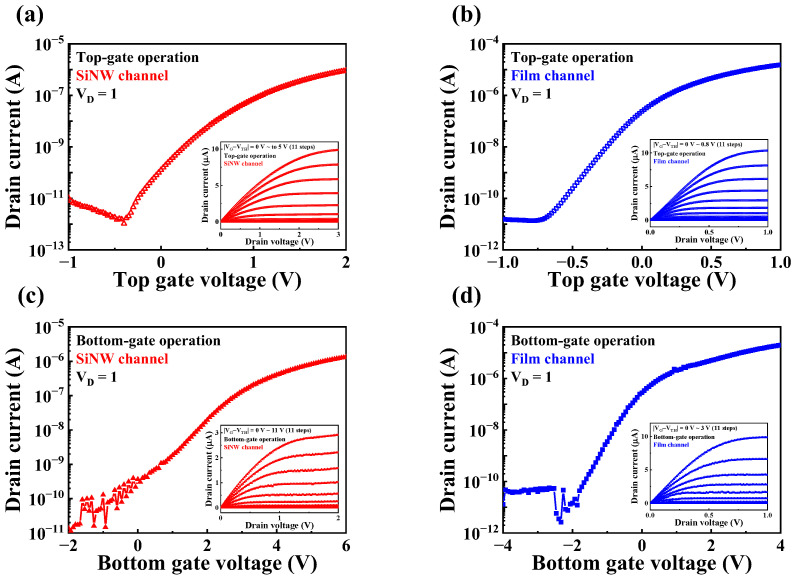
Electrical characteristics of the fabricated devices. Transfer characteristic curves for the top-gate operations of (**a**) SiNW and (**b**) film channel DG FETs, as well as the bottom-gate operations of (**c**) SiNW and (**d**) film channel DG FETs.

We fabricated FET-type sensors based on high-*k* gate dielectric engineered SiNW channel DG FETs. The operation of the fabricated FET transducer units enabled the sensing functions of the entire sensor platform. Therefore, prior to evaluating the sensing performance, the electrical characteristics of the fabricated SiNW and film channel DG FETs were compared. Figure 6 illustrates the electrical characteristics of the SiNW and film channel DG FETs. The transfer characteristic (I_DS_-V_G_) curves for the top-gate operation of the SiNW and film channel DG FETs are presented in Figure 6a,b, respectively. Meanwhile, Figure 6c,d depicts the transfer characteristic curves for the bottom-gate operations of the SiNW and film channel DG FETs, respectively. The insets show the corresponding output characteristic (I_DS_-V_D_) curves. Transfer characteristic curves were obtained at a drain voltage (V_D_) of 1 V. While sweeping either the top- or bottom-gate voltage, the other gate electrode was connected to the ground electrode. To quantitatively compare the measured electrical characteristics of the SiNW channel DG FETs with those of film channel devices, we extracted various electrical parameters, as summarized in Table 1. Although the values of the threshold voltage (V_TH_) and on/off current ratio (I_ON/OFF_) were similar, the SiNW channel DG FETs exhibited better field-effect mobility (μ_FE_) and subthreshold swing (SS) values compared to those of the film channel device. Overall, a comparison of electrical characteristics indicates that the SiNW channel DG FETs possess favorable properties for sensor applications, demonstrating improved field-effect mobility and subthreshold swing values.

### 3.2. Self-Amplification Capabilities of High-k Gate Dielectric Engineered DG FETs

**Figure 7:**
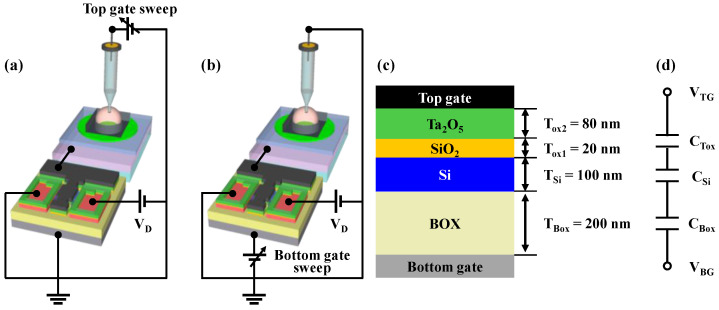
Schematic of electrical connections in (**a**) SG mode and (**b**) DG mode for the sensor platform. (**c**) Cross-sectional view of the MOSCAP structure of high-*k* gate dielectric engineered DG FET. (**d**) Schematic of the electrical equivalent circuit.

The fabricated FET devices comprised two gate electrodes: top-gate and bottom-gate electrodes. In the proposed sensor platform, the top-gate electrode is connected to the EG sensing unit, and the FETs can be operated in either SG or DG modes. Figure 7a,b depicts the electrical connections of the sensor platform in the SG and DG modes, respectively. In the SG mode (Figure 7a), only the top-gate electrode was utilized, which retained the theoretical limitation of the sensitivity associated with conventional ISFETs. However, in the DG mode (Figure 7b), the capacitive coupling between the two gate electrodes enabled the self-amplification of the sensitivity. Figure 7c shows a cross-sectional view of the metal-oxide-semiconductor capacitor (MOSCAP) structure of the high-*k* gate dielectric engineered DG FETs. The equivalent electrical circuit of the MOSCAP structure, excluding the parasitic components, is shown in Figure 7d. As shown in Figure 7d, the top-gate voltage (V_TG_) and bottom-gate voltage (V_BG_) are capacitively coupled based on the capacitances of the top-gate insulator (C_Tox_) and bottom-gate insulator (C_Box_). Because the depletion capacitance of the Si channel (C_Si_) is negligible, the relationship between ΔV_TG_ and ΔV_BG_ in the DG mode can be expressed as in Equation (1). Because the top-gate electrode was connected to the EG sensing unit, V_TG_ was equivalent to the surface potential (ψ_0_) of the analyte solution (V_TG_ = ψ_0_).

(1) $$\Delta V_{\mathrm{BG}}=\frac{C_{\mathrm{Tox}}}{C_{\mathrm{Box}}}\Delta V_{\mathrm{TG}},$$

This relationship indicates that the sensitivity of the proposed sensor platform can be amplified by the amplification factor of C_Tox_/C_Box_ due to capacitance coupling. Notably, a larger amplification factor can be achieved using a larger C_Tox_. We deliberately fabricated DG FETs with different C_Tox_ and C_Box_ values by varying the oxide thickness. To achieve a higher C_Tox_/C_Box_, we adopted a high-*k* gate dielectric engineered top-gate insulator structure by utilizing stacked oxide layers of SiO_2_/Ta_2_O_5_, which resulted in a higher C_Tox_ compared to that of single SiO_2_ oxide layers with the same thickness. Moreover, when compared to conventional film channel DG FETs, SiNW channel devices exhibit greater C_Tox_ owing to the high surface-to-volume ratio of the SiNW channel devices.

### 3.3. pH Sensing Characteristics of High-k Gate Dielectric Engineered SiNW Channel DG FETs

**Figure 8:**
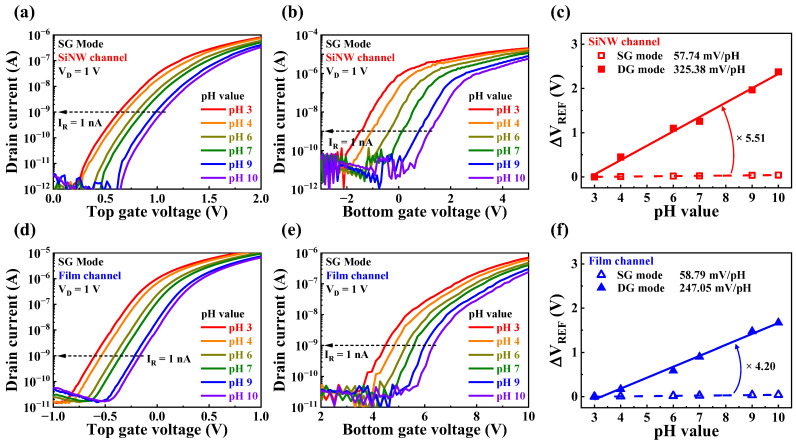
pH sensing characteristics of high-*k* gate dielectric engineered DG FETs. Transfer characteristic curves of the SiNW channel device in (**a**) SG and (**b**) DG mode with varying pH values. Transfer characteristic curves of the film channel device in the (**d**) SG and (**e**) DG mode with varying pH values. pH sensitivities of the (**c**) SiNW and (**f**) film channel devices.

The pH sensing characteristics of the high-*k* gate dielectric engineered SiNW channel DG FETs were investigated in both the SG and DG modes to verify their detection and self-amplification capabilities for the surface potential of the analyte solution. For pH sensing characterization, the SnO_2_ layer was used as the sensing membrane of the sensor platform without the formation of a Ca^2+^-selective membrane. Figure 8 illustrates the pH sensing characteristics of the fabricated sensors. The transfer characteristic curves of the SiNW channel device at various pH values in the SG and DG modes are shown in Figure 8a,b, respectively. The transfer characteristic curves of the film channel device in the SG and DG modes are shown in Figure 8d and Figure 8e, respectively. The pH sensitivities were evaluated from these transfer characteristic curves by calculating the shift in the reference voltage (V_REF_). The V_REF_ values were obtained at a read current (I_R_) of 1 nA. The calculated pH sensitivities of the SiNW and film channel devices are presented in Figure 8c,f, respectively. In the SG mode, the pH sensitivities of the SiNW and film channel devices were 57.74 and 58.79 mV/pH, respectively, demonstrating no significant difference between the SiNW and film channel DG FETs. In addition, none of the devices exceeded the Nernstian limit of 59.14 mV/pH. However, in the DG mode, the pH sensitivities of the SiNW and film channel devices were 325.38 and 247.05 mV/pH, respectively. Notably, the SiNW channel device amplified the pH sensitivity 5.51 times, whereas the film channel device amplified it 4.2 times. Because of its high surface-to-volume ratio, the SiNW channel device exhibited a higher amplification factor, resulting in a greater pH sensitivity. Thus, SiNW channel DG FETs can exhibit a pH sensing performance superior to that of conventional film channel devices.

**Figure 9:**
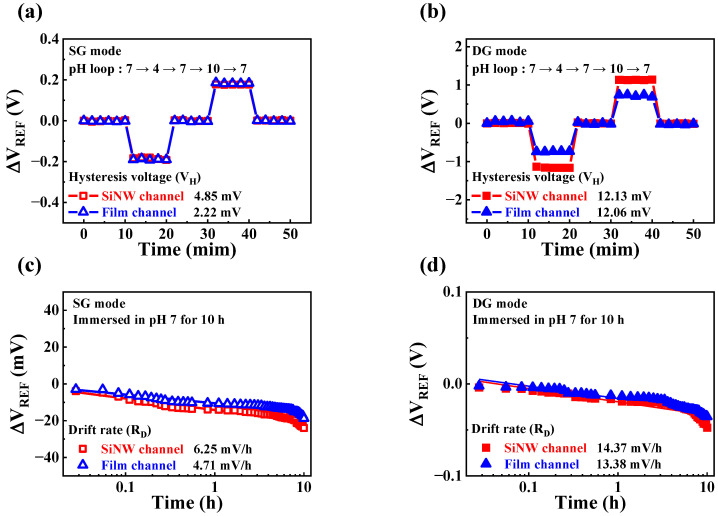
Non-ideal effects of high-*k* gate dielectric engineered DG FETs during pH sensing operations. Hysteresis effects of SiNW and film channel devices in the (**a**) SG and (**b**) DG modes. Drift effects of SiNW and film channel devices in the (**c**) SG and (**d**) DG modes.

In addition to pH sensitivity, non-ideal effects such as hysteresis and drift can affect the sensing characteristics of FET-type sensor platforms. Direct contact between the EG sensing unit and analyte solution can result in chemical damage to the sensing membrane, leading to a decrease in sensing performance. Hysteresis effects are influenced by the presence of buried OH sites and the transport of defects within the sensing membrane [31,32,33]. However, drift effects arise from the hopping or trap-limited transport of OH-related species [34,35,36,37]. Figure 9a,b displays the hysteresis effects of the SiNW and film channel DG FETs in the SG and DG modes, respectively. The hysteresis effects were evaluated by varying the pH values as 7 − 4 − 7 − 10 − 7. Transfer characteristic curves were measured every 2 min for 50 min. The hysteresis voltage (V_H_) was determined by calculating the difference between the initial and final V_REF_ values. In the SG mode, the resulting V_H_ values for SiNW and film channel devices were 4.85 and 2.22 mV, respectively, while in DG mode, the corresponding values were 12.13 and 12.06 mV, respectively. The drift effects of the SiNW and film channel DG FETs in the SG and DG modes are depicted in Figure 9c,d, respectively. The drift rate (R_D_) was measured after the sensing membrane was immersed in a pH 7 buffer solution for 10 h. The fabricated SiNW and film channel devices exhibited R_D_ values of 6.25 and 4.71 mV/h, respectively, in the SG mode. In the DG mode, the corresponding values were 14.37 and 13.38 mV/h, respectively. Table 2 presents the pH sensing characteristics of the fabricated devices, including pH sensitivity, V_H_, R_D_, V_H_-to-pH sensitivity, and R_D_-to-pH sensitivity. The results indicated that the DG mode operation yielded higher values for both V_H_ and R_D_, as well as pH sensitivity, compared to the SG mode for both the SiNW and film channel devices. However, when considering the V_H_-to-pH sensitivity and R_D_-to-pH sensitivity, the increase in the V_H_ and R_D_ values was notably lower than the increase in pH sensitivity for each device. Furthermore, the SiNW channel device exhibited a more significant reduction in both V_H_-to-pH sensitivity and R_D_-to-pH sensitivity in the DG mode compared to the film channel device. These findings suggest that capacitive coupling in the DG mode is an effective approach for amplifying the sensitivity beyond the theoretical limit, resulting in higher sensitivity, stability, and reliability. Consequently, the proposed high-*k* gate dielectric engineered SiNW channel DG FET demonstrates promising potential as a high-performance sensor platform, offering highly sensitive and stable sensing characteristics.

### 3.4. Ca^*2*+^-Selective Sensing Characteristics of the High-k Gate Dielectric Engineered SiNW Channel DG FETs

**Figure 10:**
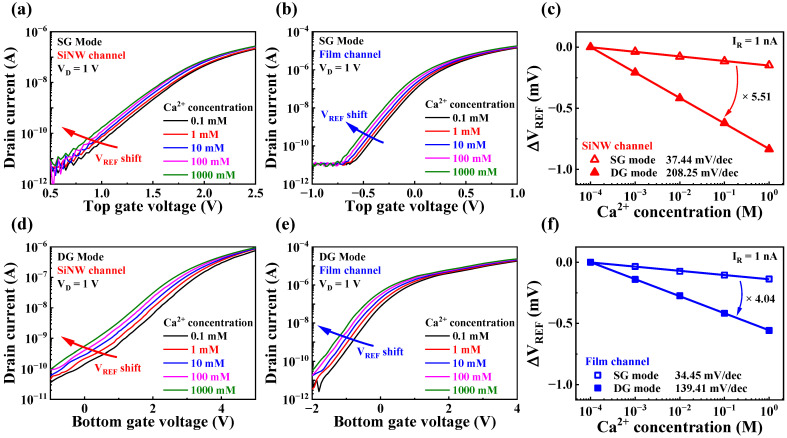
Ca^2+^ sensing characteristics of high-*k* gate dielectric engineered DG FETs. Transfer characteristic curves of the SiNW channel device in the (**a**) SG and (**b**) DG modes with varying Ca^2+^ concentrations. Transfer characteristic curves of the film channel device in the (**d**) SG and (**e**) DG modes with varying Ca^2+^ concentrations. Ca^2+^ sensitivities of the (**c**) SiNW and (**f**) film channel devices.

After successfully demonstrating the high-performance sensing capabilities of the fabricated high-*k* gate dielectric engineered SiNW channel DG FET sensor platform, we applied our device to practical biosensing applications, specifically, the selective detection of Ca^2+^. To enable the device as a Ca^2+^-selective sensor, we fabricated a Ca^2+^-selective EG by forming a Ca^2+^-selective membrane on a SnO_2_ layer. The detailed fabrication process of the Ca^2+^-selective EG sensing unit is described in Section 2.4. Figure 10a,b presents the transfer characteristic curves of the SiNW channel devices in the SG and DG modes, respectively, with varying Ca^2+^ concentrations. The corresponding results for the film channel devices are shown in Figure 10d,e, respectively. The transfer characteristic curves were measured using CaCl_2_ solutions with varying Ca^2+^ concentrations. As the Ca^2+^ concentration increased, the transfer characteristic curves shifted in the negative direction. Figure 10c,f show the Ca^2+^ sensitivities of the SiNW and film channel devices, respectively. The V_REF_ values were obtained at an I_R_ of 1 nA. In the SG mode, the Ca^2+^ sensitivities of the SiNW and film channel devices were 37.44 and 34.45 mV/dec, respectively. In the DG mode, the corresponding Ca^2+^ sensitivities were 208.25 and 139.41 mV/dec, respectively. While both devices exhibited similar Ca^2+^ sensitivities in the SG mode, the Ca^2+^ sensitivities of the SiNW and film channel devices were amplified by factors of 5.51 and 4.04, respectively, in the DG mode. This result is consistent with the findings observed in the pH sensing operations described in Section 3.3. Therefore, the fabricated Ca^2+^ sensor based on the high-*k* gate dielectric engineered SiNW channel DG FET demonstrated highly sensitive characteristics, suggesting its potential for various biosensing applications.

**Figure 11:**
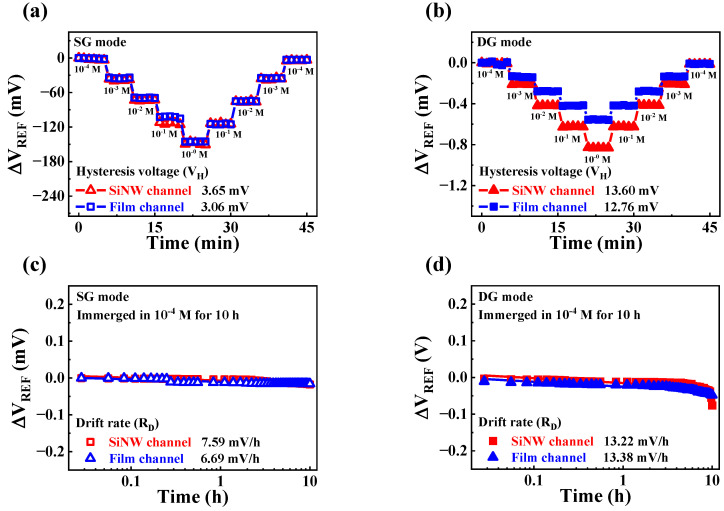
Non-ideal effects of high-*k* gate dielectric engineered DG FETs with Ca^2+^ sensing operations. Hysteresis effect of high-*k* gate dielectric engineered DG FETs in the (**a**) SG and (**b**) DG modes. Drift effects of high-k gate dielectric engineered DG FETs in the (**c**) SG and (**d**) DG modes.

To verify the stability and reliability of the fabricated Ca^2+^ sensor, we conducted hysteresis and drift effect measurements during the Ca^2+^ sensing operations. Figure 11a,b shows the hysteresis effects of the SiNW and film channel devices with CaCl_2_ solutions in the SG and DG modes, respectively. We measured the hysteresis effects of the Ca^2+^ sensing operations for 45 min, changing the Ca^2+^ concentration every 5 min according to the following CaCl_2_ concentration loop: 10^−4^ − 10^−3^ − 10^−2^ − 10^−1^ − 10^0^ − 10^−1^ − 10^−2^ − 10^−3^ − 10^−4^ M. V_REF_ values were extracted from the transfer characteristic curves measured every 1 min. In the SG mode, the V_H_ values of SiNW and film channel devices were 3.65 and 3.06 mV, respectively. In the DG mode, the corresponding values were 13.60 and 12.76 mV, respectively. Figure 11c,d shows the drift rates of the SiNW and film channel devices, respectively, for Ca^2+^ operation. The R_D_ values of the SiNW and film channel devices were monitored for 10 h, while the Ca^2+^-selective membrane of EG was immersed in a 10^−4^ M CaCl_2_ solution. In the SG mode, the R_D_ values of SiNW and film channel devices were 7.59 and 6.69 mV/h, respectively. In the DG mode, the corresponding values were 13.22 and 13.38 mV/h, respectively. Table 3 summarizes the Ca^2+^-sensing characteristics of the high-*k* gate dielectric engineered DG FETs. Although the V_H_ and R_D_ values increased in the DG mode compared to the SG mode, the increase in these non-ideal effects was much smaller than the increase in the Ca^2+^ sensitivity of both devices. Moreover, in the DG mode, the SiNW channel device significantly reduced the V_H_ and R_D_ to Ca^2+^ sensitivity from 9.74% to 6.65% and 20.27% to 6.34%, respectively. The observed enhancement in stability is consistent with the pH sensing results and is attributed to the high amplification capability of the SiNW channel device. Thus, we verified the stable Ca^2+^ sensing characteristics of the fabricated high-*k* gate dielectric engineered SiNW channel DG FETs.

**Figure 12:**
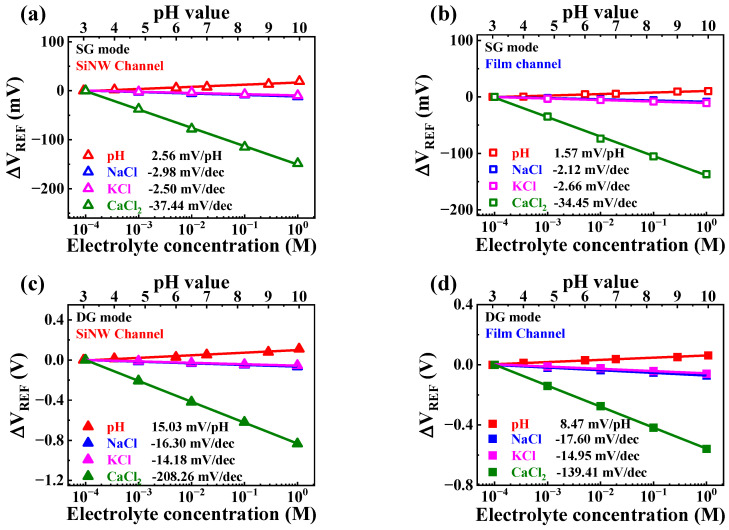
Ca^2+^-selective sensing characteristics of high-*k* gate dielectric engineered DG FETs. Various ion (H^+^, Na^+^, K^+^, and Ca^2+^) sensitivities of (**a**) SiNW channel device in SG mode, (**b**) film channel device in SG mode, (**c**) SiNW channel device in DG mode, and (**d**) film channel device in DG mode.

To establish Ca^2+^-selective sensing characteristics, we measured the pH, Na^+^, and K^+^ sensitivities of Ca^2+^-selective EG using pH buffer, NaCl, and KCl solutions, respectively. Figure 12a,b depicts the Ca^2+^-selective sensing characteristics of the SiNW and film channel devices, respectively, in the SG mode. Among the interfering ions, including H^+^, Na^2+^, and K^+^, the highest interfering-ion sensitivities were only 7.95% and 7.72% for the Ca^2+^ sensitivities of the SiNW and film channel devices, respectively. Figure 12c,d shows the Ca^2+^-selective sensing characteristics of the SiNW and film channel devices, respectively, in the DG mode. The maximum interfering-ion sensitivities measured in the DG mode were 7.82% and 12.62% for the Ca^2+^ sensitivities of the SiNW channel and film channel devices, respectively. The interfering-ion sensitivities are assumed to be amplified along with the Ca^2+^ sensitivities, proportional to the amplification factor, as the interfering-ion sensitivity arises from the transfer of the ion’s surface potential (ψ_0_) to the sensing membrane [38,39,40,41]. However, considering that the interfering-ion sensitivity in the DG mode for the SiNW channel device was limited to less than 7.82% of the Ca^2+^ sensitivity, this corresponds to a negligible value that does not significantly hinder the selective Ca^2+^ sensing operation. Therefore, these findings suggest that the fabricated Ca^2+^-selective sensor, constructed with high-*k* gate dielectric engineered SiNW channel DG FETs, exhibits extensive versatility as a high-performance biosensor platform, owing to its ultrasensitive and highly selective sensing characteristics for the selective detection of Ca^2+^. Table 4 summarizes the pH, Na^+^, K^+^, and Ca^2+^ sensing characteristics of the high-*k* gate dielectric engineered DG FETs.

## 4. Conclusions

In this study, we present a high-performance biosensor platform based on high-*k* gate dielectric engineered SiNW random network channel DG FETs for the selective detection of Ca^2+^. The proposed sensor platform combines the advantages of high-*k* gate dielectric engineered SiNW channel DG FETs as transducer units and separate EG as a sensing unit. The template transfer method using PVP nanofibers enabled the fabrication of SiNW channels. Due to the high surface-to-volume ratio of the SiNW channel structure, top-gate oxide capacitance of the SiNW channel device could be larger, thereby enhancing the self-amplification capability of capacitively coupled DG FETs. In addition to the SiNW channel device, a conventional film channel device was fabricated to validate the improved characteristics of the sensor platform. The electrical characteristics and pH-sensing capabilities of the sensor platform were thoroughly evaluated to lay the foundation for Ca^2+^ detection. The integration of a Ca^2+^-selective membrane to the fabricated sensor platform resulted in remarkable Ca^2+^ sensitivity, with the SiNW channel device achieving a sensitivity of 208.25 mV/dec, surpassing that of the film channel device by 149%. The assessment of non-ideal effects, such as hysteresis and drift, demonstrated that the fabricated SiNW channel device effectively mitigated these effects, with the V_H_ and R_D_ values remaining below 6.53% despite the enhanced Ca^2+^ sensitivity. To further assess its selective sensing capabilities, the sensitivity of the platform to interfering ions, including H^+^, K^+^, and Na^+^, was evaluated using a pH buffer, KCl, and NaCl solutions. Although the interfering-ion sensitivities were also amplified in proportion to the Ca^2+^ sensitivity, the SiNW channel device exhibited a sensitivity of less than 7.82% of the amplified Ca^2+^ sensitivity. These results confirm the successful application of the proposed sensor platform as a high-performance biosensor. Therefore, the proposed high-performance biosensor platform based on high-*k* gate dielectric engineered SiNW random network channel DG FETs demonstrated highly sensitive and selective characteristics with reliable sensing operation. These exhibit promising potential for broad applications in various biosensing fields, highlighting applicability and versatile capabilities in biomedical diagnostics, environmental monitoring, and food safety analysis.

## Figures and Tables

**Table 1:** Electrical parameters evaluated from the transfer characteristic curves, including threshold voltage (V_TH_), on/off current ratio (I_ON/OFF_), field-effect mobility (μ_FE_), and subthreshold swing (SS).

Operating Electrode	Channel Type	V_TH_ (V)	I_ON/OFF_ (A/A)	μ_FE_ (cm^2^/V·s)	SS (mV/dec)
Top gate	SiNW	−0.5	2.7 × 10^6^	308.6	136.1
	Film	−0.8	1.1 × 10^6^	280.91	144.2
Bottom gate	SiNW	−1.5	1.7 × 10^5^	159.6	172.1
	Film	−2.4	7.4 × 10^5^	134.2	181.7

**Table 2:** pH sensing characteristics of high-*k* gate dielectric engineered DG FETs, including pH sensitivity, hysteresis voltage (V_H_), drift rate (R_D_), V_H_-to-pH sensitivity, and R_D_-to-pH sensitivity.

Operation Mode	Channel Type	pH Sensitivity (mV/pH)	V_H_ (mV)	R_D_ (mV/h)	V_H_-to-pH Sensitivity	R_D_-to-pH Sensitivity
SG mode	SiNW	57.74	4.85	6.25	8.3%	10.82%
	Film	58.79	2.22	4.71	3.77%	8.01%
DG mode	SiNW	325.38	12.13	14.37	3.72%	4.41%
	Film	247.05	12.06	13.38	4.88%	5.41%

**Table 3:** Ca^2+^ sensing characteristics of high-*k* gate dielectric engineered DG FETs.

Operation Mode	Channel Type	Ca^2+^ Sensitivity (mV/dev)	V_H_ (mV)	R_D_ (mV/h)	V_H_-to-Ca^2+^ Sensitivity	R_D_-to-Ca^2+^ Sensitivity
SG mode	SiNW	37.44	3.65	7.59	9.74%	20.27%
	Film	34.45	3.06	6.69	8.88%	19.41%
DG mode	SiNW	208.25	13.60	13.22	6.53%	6.34%
	Film	139.41	12.76	13.38	9.15%	9.59%

**Table 4:** Summary of the pH, Na^+^, K^+^, and Ca^2+^ sensing characteristics of high-*k* gate dielectric engineered DG FETs.

Operation Mode	Channel Type	pH Sensitivity (mV/pH)	Na^+^ Sensitivity (mV/dec)	K^+^ Sensitivity (mV/dec)	Ca^2+^ Sensitivity (mV/dec)
SG mode	SiNW	2.56	2.98	2.50	37.44
	Film	1.57	2.12	2.66	34.45
DG mode	SiNW	15.03	16.30	14.18	208.25
	Film	8.47	17.60	14.95	139.41

## Data Availability

Not applicable.
